# Vasculogenic dynamics in 3D engineered tissue constructs

**DOI:** 10.1038/srep17840

**Published:** 2015-12-09

**Authors:** Yaron J. Blinder, Alina Freiman, Noa Raindel, David J. Mooney, Shulamit Levenberg

**Affiliations:** 1 Department of Biomedical Engineering, Technion – Israel Institute of Technology, Haifa, Israel.; 2 School of Engineering and Applied Sciences, Harvard University, Cambridge, MA 02138, United States.; 3 Wyss Institute for Biologically Inspired Engineering, Harvard University, Cambridge, MA 02138, United States.

## Abstract

Implantable 3D engineered vascular tissue constructs can be formed by co-culturing endothelial and fibroblast cells on macroporous scaffolds. Here we show that these constructs can be used for studying the dynamics of neovascular formation *in-vitro* by a combination of live confocal imaging and an array of image processing and analysis tools, revealing multiple distinct stages of morphogenesis. We show that this process involves both vasculogenic and angiogenic elements, including an initial endothelial multicellular cluster formation followed by rapid extensive sprouting, ultimately resulting in a stable interconnected endothelial network morphology. This vascular morphogenesis is time-correlated with the deposition and formation of an extensive extra-cellular matrix environment. We further show that endothelial network junctions are formed by two separate morphogenic mechanisms of anastomosis and cluster thinning.

The viability and clinical success of implantable tissue constructs depend on the successful integration of the construct into the host vascular system. For this reason, vascularization is widely recognized as a fundamental issue in regenerative medicine and tissue engineering research^1^,^2^. Various methods have been reported for studying vascular morphogenesis in 3D systems^1^,^3^,^4^,^5^. These methods include *in-vivo* models such as zebrafish^6^ and the chick chorioallantoic membrane^7^, *ex-vivo* assays such as aortic ring explants, and *in-vitro* systems including co-cultured endothelial cell and pericytes suspended in collagen^8^, embryoid bodies suspended in collagen^9^ and EC-coated microcarrier beads suspended in fibrin^10^. Recently there have also been several developments in microfabricated systems for vascular research^11^,^12^,^13^,^14^,^15^. We have previously demonstrated that formation of a preliminary vascular network within an engineered tissue construct prior to implantation (prevascularization) improved host integration^16^,^17^,^18^,^19^,^20^.

Current understanding in vascular biology categorizes mechanisms of new blood vessel formation *in-vivo* into two major categories – vasculogenesis and angiogenesis. Vasculogenesis occurs mainly in the developing embryo, and in some cases during adult wound healing (where it is referred to as “adult vasculogenesis”^21^,^22^), and entails the recruitment of endothelial progenitor cells (angioblasts in the embryo, circulating bone marrow-derived progenitors in the adult^21^,^22^) which interact with matrix-producing mesenchymal cells to form a vascular plexus *de-novo*. Angiogenesis is the formation of new blood vessels via sprouting or branching from pre-existing vessels. This sprouting mechanism is typically instigated by a pro-angiogenic signal such as a local gradient of VEGF (e.g. as a response to hypoxia^23^,^24^,^25^). In the presence of such a signal, endothelial cells from an existing blood vessel break apart their basement membrane by secreting matrix metalloproteases (MMPs), and begin a series of events known as “tip-cell selection” – a NOTCH-DLL4-dependent process resulting in the selection of a “tip-cell” which leads the sprouting process, sensing its surroundings with filopodia and supported from behind by endothelial “stalk-cells”^9^,^26^,^27^. If successful, angiogenic sprouts anastomose with other existing blood vessel and support blood flow.

While much research has been published on these different mechanisms dominating vascular morphogenesis both *in-vivo* and *in-vitro*, the *in-vitro* systems are generally optimized for ease of analysis and are not clinically applicable. Additionally, the formation of new vascular networks in implantable 3D engineered tissue has not been studied in real-time, and the dynamics of its underlying mechanisms have not been fully characterized.

In this paper, we present the novel use of 3D engineered tissue constructs as a research modality for the study of vascular morphogenesis *in-vitro*. By combining live imaging and analysis of the morphological processes of neovascular network formation in 3D engineered tissue constructs, we are able to characterize new mechanisms of network junction formation.

## Results

### Endothelial and fibroblast morphogenesis during neovascularization in 3D engineered tissue constructs

**Figure 1:**
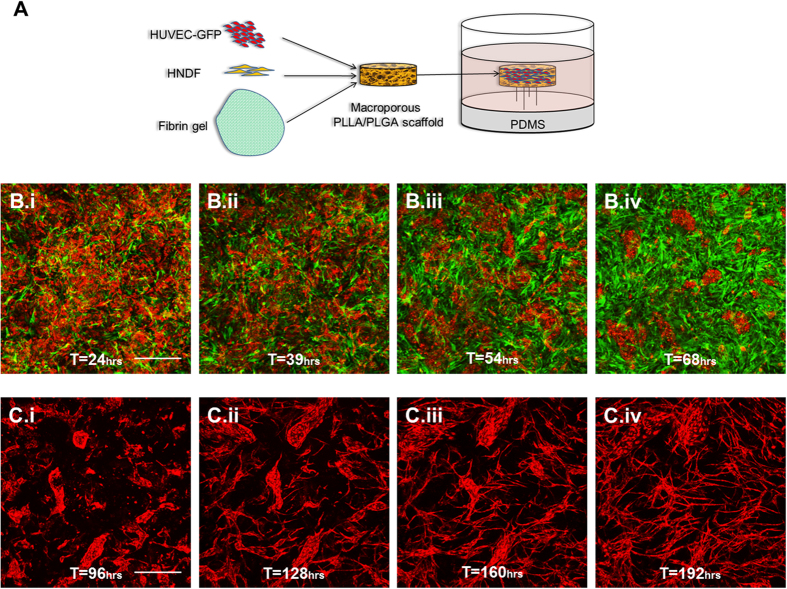
Endothelial and fibroblast morphogenesis during neovascularization. (**A**) Schematic representation of the seeding process. (**B**.i-B.iv) HUVEC (red) and HNDF (green) morphogenesis over days 1–3 post-seeding (scale bar = 500 um). (**C**.i-C.iv) HUVEC morphogenesis over days 4–7 post-seeding (scale bar = 500 μm).

Implantable 3D vascularized engineered tissue can be constructed by co-culturing endothelial cells (ECs) and fibroblast cells (FCs) on a macroporous scaffold^16^,^17^,^18^,^19^,^20^. To study the different roles and morphological processes of each cell type in such a system we first seeded fluorescently labeled cells and fixed the scaffolds on pins to enable live imaging over time (Fig. 1A). Once seeded on the scaffold, ECs and FCs exhibited markedly different behaviors. FCs began to undergo massive proliferation almost immediately, rapidly filling the entire volume of the scaffold over a period of roughly 4 days (Fig. 1B). Unlike the FCs, ECs underwent a multi-stage morphogenic process. During the first 4 days, ECs migrated and formed multicellular clusters. These clusters then began to exhibit outward sprouting, resulting in the formation of a branched endothelial network around day 7 (Fig. 1B,C, supplementary movies S1, S2). When seeded without the presence of fibroblast cells in the scaffold, endothelial cells exhibited little to no vasculogenic behavior, maintaining instead their initial random distribution throughout the construct over time (Figure S1).

### Endothelial tip cell dynamics

**Figure 2:**
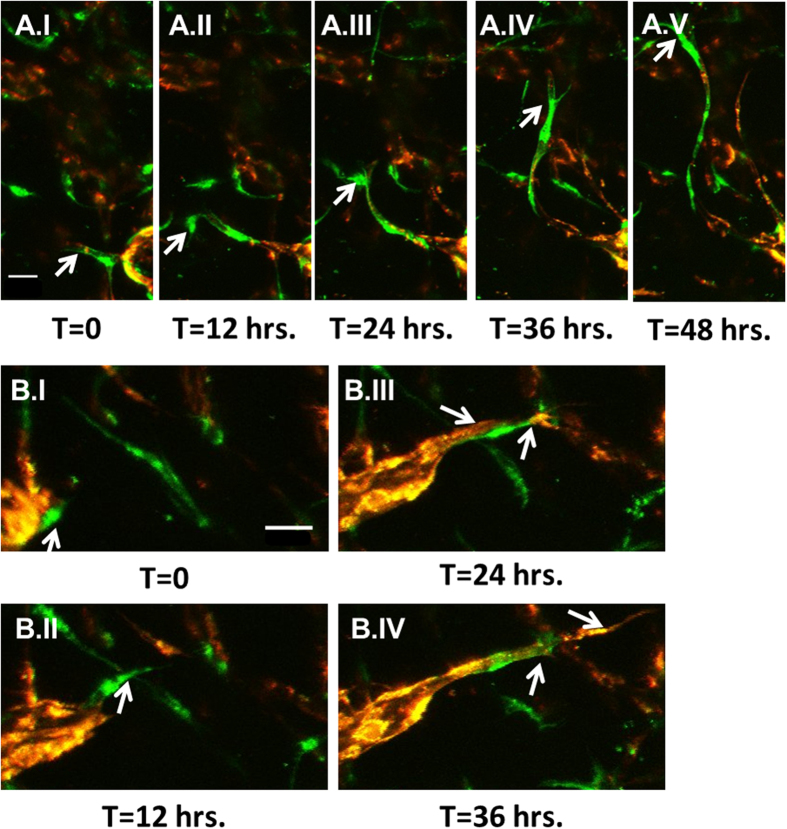
Endothelial sprouts are led by dynamically competing tip cells. (**A**.I-A.V) Time lapse imaging of a tip cell in an individual sprout. (**B**.I-B.IV) RFP- and GFP- expressing HUVECs show cells switching at the tip cell position. (scale bars = 100 μm)

Endothelial sprouts within the constructs exhibited a “tip-cell” at their leading edge with visible filopodia extruding outward prior to moving forward (Fig. 2A, supplementary movie S3). By using a mixture of GFP- and RFP-expressing HUVECs these tip-cells can be seen alternating with their supporting “stalk-cells” (Fig. 2B, supplementary movie S4) during the sprouting process, in a similar fashion to previously characterized “dynamic competition”^28^.

**Figure 3:**
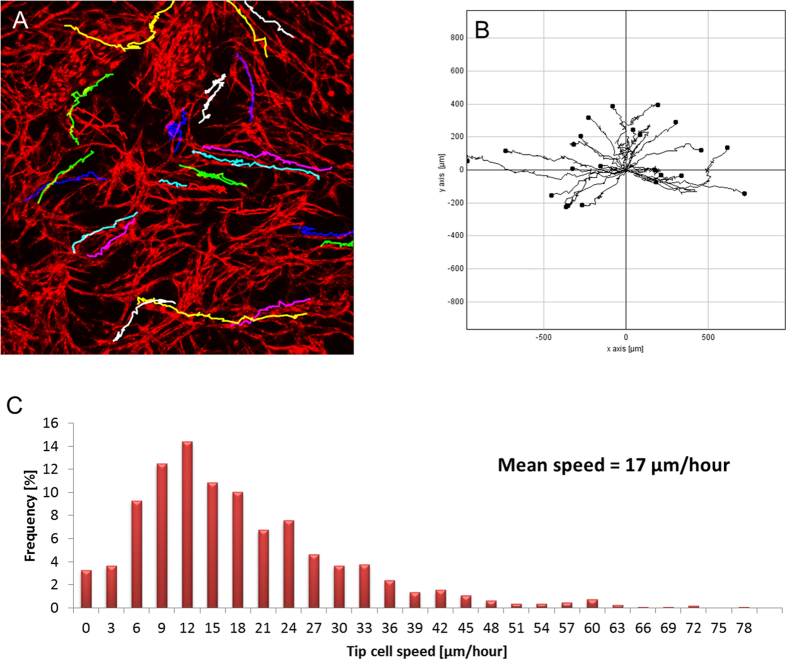
Tip cell speed measurement by manual tracking. (**A**) Example of manually tracked tip cell trajectories. (**B**) Multiple tip cell trajectories plotted from a shared origin point. (**C**) Histogram of tip cell velocities calculated from manual tracking. Number of trajectories = 25. Average velocity = 0.281 [μm/min].

To learn more about the dynamics of vessel sprouts in this assay we manually tracked tip cell positions in individual sprouts over time. Sprout directionality and speed distribution analysis showed endothelial sprouting speed averaged ~17 μm/hr with no clear directional preference for sprouting (Fig. 3).

### Different mechanisms of network junction formation

**Figure 4:**
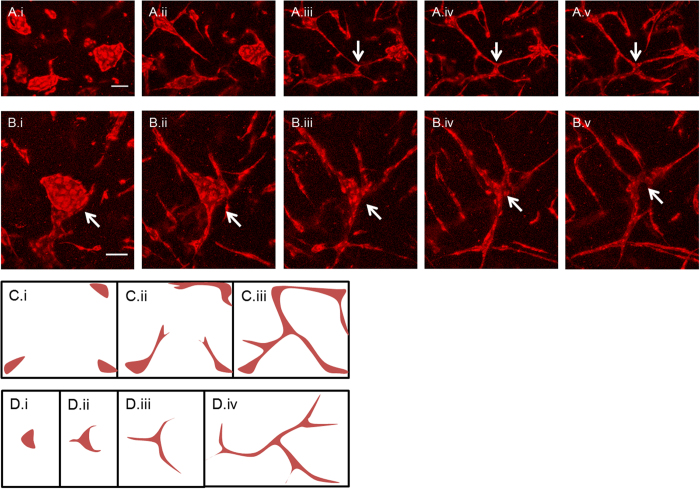
Different mechanism of junction formation. (**A**.i-A.v) Junction formation by anastomosis of multiple disparate sprouts. (**B**.i-B.v) Junction formation by cluster thinning. (scale bars = 100 μm). (**C**.i-C.iii) Illustration of junction formation by sprout anastomosis. (**D**.i-D.iv) Illustration of junction formation by cluster thinning.

The formation of vascular network junctions within engineered tissue constructs has not been previously described. Time-lapse confocal microscopy of spatially-fixed constructs revealed two very different morphological processes by which endothelial cells formed these junctions. Some network junctions were formed when endothelial sprouts from separate clusters met and anastomosed to form a stable connection or junction; alternatively, network junctions were formed following sprouting in multiple directions from a single cluster, combined with that cluster diminishing in size throughout the sprouting process, leaving behind stable junctions (Fig. 4).

### Image analysis-based quantification of endothelial network morphogenesis

**Figure 5:**
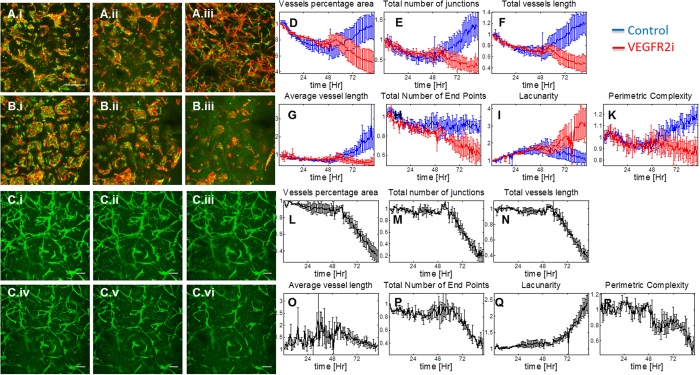
Quantification of network morphogenesis. (**A**.i-A.iii, **B**.i-B.iii) Time points visualization of RFP- and GFP-expressing HUVECs during neovascular morphogenesis. Group A is the control, group B is treated with SU5416 at day 5. T = 3 days, 5 days and 7 days post-seeding, respectively. Scale bar = 500 μm. (**C**.i-C.vi) Time points visualization of a mature (14 day old) vascular construct, treated with SU5416 after 48 hrs. T = day 14 + 0, 17 hrs, 34 hrs, 51 hrs, 68 hrs and 85 hrs, respectively. Scale bar = 200 μm. D-K, L-R: Quantification graphs for morphological parameters: vessel percentage area, total number of junctions, total vessels length, Average vessel length, total number of end points, lacunarity and perimetric complexity, respectively. Results are normalized to T = 0.

In order to quantify the network-forming processes in the time-lapse data, we employed two sets of image processing and analysis tools. Firstly, we implemented proprietary Matlab code to extract a morphological metric previously defined as “perimetric complexity”^29^ (supplementary Figure S2), which quantifies how different a shape is from a circle – the simplest geometric shape. We then applied this metric to each connected component in each frame of the time-lapse data (Fig. 5K). Secondly, we used “Angiotool” (NCI) - a freeware image analysis tool designed specifically for quantification of neovascular structures^30^, which enables the extraction of characteristic morphological parameters from an image of a vascular network. Analyzing over a period of 4 days starting at day 3 post-seeding, this analysis showed that vessel percentage area, total number of junctions, total vessels length, and perimetric complexity all followed a similar pattern of decrease during days 3–5 (during the clustering phase) followed by a significant increase in days 5–7 (during the sprouting and maturation phase). This pattern was reversed for the “lacunarity” (a metric of vessel non-uniformity) obtained with Angiotool, and the total number of endpoints showed a slight initial decrease (<10%) followed by stabilization.

### Effect of VEGFR2 inhibition on developing and matured vascular networks in engineered constructs

Inhibition of VEGFR2 has been recognized in recent years as an interesting target for antiangiogenic cancer therapies, resulting in a number of molecules designed specifically for this purpose^31^. In order to visualize the effect of such inhibition on engineered microvascular networks, scaffolds were subjected to culture media supplemented with SU5416 (Semaxanib) and imaged in real time. Four day old and 14 day old constructs were used to study the effect of VEGFR2 inhibition on developing as well as more matured neovascular networks.

VEGFR2 inhibition in developing endothelial networks resulted in a complete halting of endothelial sprouting as well as an acute regression of all active sprouts, and a reversal of the morphogenic trends discussed earlier (Fig. 5D,K).

Prior to inhibition, endothelial network morphology in mature engineered tissue constructs (past day 7) continued to progress, and stabilized by day 14. Time-lapse imaging of 14-day-old engineered tissue constructs showed little vascular remodeling over time. However, when subjected to selective inhibition of VEGFR2, these same structures showed significant sprout regressions and vessel pruning. Similarly, specific morphological parameters such as vessel percentage area, total number of junctions, total and average vessel lengths and total number of endpoints followed a similar pattern of stability followed by VEGFR2 inhibition-related decrease. The lacunarity metric showed a mirrored behavior, where stability was followed by a VEGFR2 inhibition-related increase. (Fig. 5L–R, supplementary movie S9)

### Deposition of ECM proteins

To further study the role of fibroblast cells in engineered tissue constructs, we examined the deposition of secreted extra-cellular matrix (ECM) proteins. xCollagen I production and deposition was observed as early as day 1 post-seeding, and increased over the first 5 days correlatively with the proliferation of the fibroblast population (Supplementary Figure 3). By day 5, the scaffolds contained an extracellular environment laden throughout with various common ECM proteins such as fibrous Collagen I and Laminin. Collagen IV could be seen co-localized with endothelial vessels (Supplementary Figures S4-S5, supplementary movie S10).

## Discussion

Vascularization of implantable engineered tissue constructs is crucial to viability and consequent clinical success of the constructs post-implantation. An in-depth understanding of the neovascular formation process is therefore highly significant for the optimization of tissue engineering-related therapeutic approaches. A wide variety of excellent assays have been developed for the purpose of studying endothelial behavior during neovascular formation. These *in-vitro* vascular morphogenesis assays generally focus on angiogenic sprouting from an existing surface or pre-formed vessel^1^,^3^,^4^,^5^,^9^,^10^,^11^,^12^,^13^,^14^,^15^, and do not typically exhibit endothelial clustering prior to sprouting. A previously reported 3D vasculogenesis assay^8^ consisting of co-cultured endothelial cells and pericytes in a collagen gel shows endothelial elongation and network formation *de novo* with no prior clustering. This may be due to the cell seeding density, which is more than a full order of magnitude lower than in our assay. As clustering effectively lowers local endothelial density, this may suggest a feedback mechanism.

When compared to *in-vivo* processes of neovascular morphogenesis, endothelial behavior in our assay is closely reminiscent of developmental neovascular formation which is characterized by vasculogenesis followed by angiogenesis. Similarly, our system could be said to recapitulate adult vasculogenesis, where EPCs initially form clusters in the interstitial spaces of a wound before eventually sprouting to integrate with the local blood vessels^22^.

**Figure 6:**
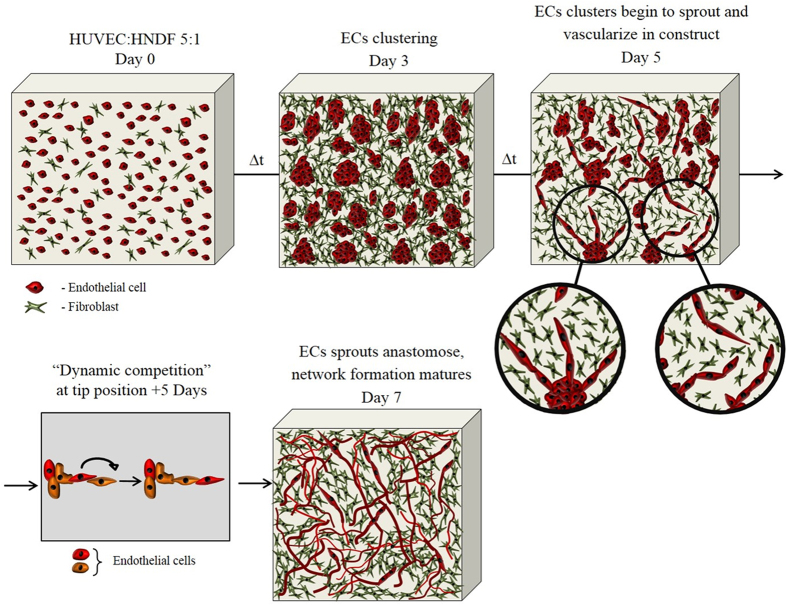
Schematic representation of multi-stage neovascular formation.

In this study we used engineered vascular tissue constructs in a novel approach to study the development of new blood vessels in this context. Using live confocal microscopy and an array of image processing and analysis tools we show that neovascular formation in engineered tissue constructs occurs through a multi-stage morphogenic process. Initially, randomly distributed endothelial cells migrate and form multicellular clusters, concurrent with fibroblast proliferation and deposition of ECM proteins. Subsequently the endothelial clusters undergo extensive sprouting, and network junctions begin to form by a combination of sprout anastomoses and thinning of clusters, resulting in developed microvascular network morphology within ~1 week (Fig. 6). We have shown in earlier work^17^ that endothelial cluster formation occurred even when the cell density was one-third the density used in this work. Endothelial:Fibroblast ratio was a determining factor in successful neovascular morphogenesis, with a 5:1 ratio producing optimal results.

When cultured on tissue engineering scaffolds, endothelial cells alone do not form this network morphology. Previous studies have shown that a second, mesenchymal-derived cell population is required in order for endothelial network morphogenesis to occur, a fact generally attributed to the mesenchymal-derived cells’ ability to take on a mural cell phenotype^32^. The results show that these cells also play an important role in the deposition of a significant and varied extra-cellular matrix surrounding the nascent blood vessels. The time-scale correlation between this matrix deposition and endothelial morphogenesis in our assay suggests this matrix may play a role in promoting endothelial network formation. Of the materials used in this assay, the fibrin hydrogel gel most immediately and directly affects cell organization post-seeding. Fibrin hydrogel is very similar both mechanically and biochemically to native ECM, and indeed serves as a natural scaffold in early wound healing. Macroporous PLGA provides mechanical support to the seeded cell-fibrin composite, enabling the construct to retain its 3D shape for an extended period of time^17^.

Real-time microscopy enables not only morphological analysis throughout the sprouting process, but also characterization and measurement of dynamic parameters such as sprout directionality and speed. The measured average sprouting speed value is a full order of magnitude higher than those previously reported for EB assays^28^ and on par with those reported in zebrafish angiogenesis^33^ assays, possibly as a result of the supporting structure and composition of the deposited ECM. The different mechanisms of network junction formation visualized in our assay are previously unreported, highlighting the value of this type of assay for exploratory research. Additionally, this method enables direct visualization and time-course dynamics analysis of the effect of certain drugs on the engineered microvascular network, as we showed by selectively inhibiting VEGFR2. This exemplifies the potential of this method to be used in future drug screening and development.

## Methods

### Cell culture

GFP- or RFP-expressing Human Umbilical Vein Endothelial Cells (HUVECs) (passage 4–6, Angioproteomie) were cultured on tissue culture flasks in EGM-2 medium supplemented with FBS (5% total FBS). Neonatal Human Dermal Fibroblasts (HNDFs) (passage 4–6, Clonetics) were cultured on tissue culture flasks in Dulbecco’s Modified Eagle’s Medium (DMEM, Gibco) supplemented with 10% FBS (Hyclone), 1% non-essential amino acids (NEAA), 1% pen-strep and 0.2% b-mercaptoethanol (Sigma Aldrich).

### Vascular construct preparation

Macroporous PLLA/PLGA scaffolds were prepared using a porogen leaching protocol as previously described^17^. 3D engineered vascular constructs were prepared using a modified protocol. Briefly, HUVECs and HNDFs were combined (500,000 and 100,000 per scaffold, respectively) and reconstituted within a solution containing fibrinogen (7.5 mg/ml, Sigma) and Thrombin (10 U/ml, Sigma). The mixture was immediately seeded onto the macroporous scaffolds and incubated at 37 C for 1 hour before addition of culture media. The media used was equal parts HUVEC and HNDF media as described in the cell culture section above.

### VEGFR2 inhibition

To selectively inhibit VEGFR2, scaffolds were subjected to culture medium supplemented with 20 μM of the tyrosine kinase inhibitor SU5416 (Sigma-Aldrich).

### Time-lapse microscopy

1 ml of PDMS mixture (SYLGARD® 184) was added to each well in a 24-well plate. 3 thin metal pins (insect pins, Austerlitz) were positioned at equidistant (~1–2 mm) points from the center of each well and the PDMS was allowed to harden by heating to 80 °C for 30 min. Prior to initiation of each time-lapse protocol, scaffolds were placed on the pins in each of the scaffold-holder wells and 2 ml of cell culture media were added to each well. The multiwall plate was then placed inside the incubation chamber of an upright LEICA SuperZoom Z6 confocal microscope at 37 °C, with 5% CO2. For each scaffold, an optimal initial position was chosen based on apparent local HUVEC population morphology, and a z-stack was defined to capture the maximum field-of-view in the z-axis. A time-series was then registered at 1 frame/hour for each scaffold. For time-lapse experiments longer than 48 hours, the time-series acquisition was halted after 48 hours for media change, then the z-stacks were redefined at each position to correct for any motion in the z-axis that the scaffolds may have had as a result of the media change.

### Immunohistochemical staining

Preparation of whole-mount and scaffold cross-sections for staining included fixation in 4% paraformaldehyde in PBS, followed by permeabilization in a 1% Triton solution, washing and blocking with a BSA-supplemented staining buffer (BD Pharmingen). Samples were then incubated with primary antibodies for Collagen I (Abcam), Laminin (Chemicon), Collagen IV (Dako) and CD31 (BD Pharmingen) overnight, followed by several washes in PBS, 3-hour incubation with secondary antibodies in staining buffer (Pharmingen) and final washing steps before imaging.

### Image processing and analysis

4-D (XYZT) confocal Z-stacks were converted to 2-D time-series TIFF stacks by performing z-projections at each time step using the NIH ImageJ software. Stacks were then prepared for morphological analysis by contrast enhancement (stack histogram equalization and normalization, 0.4% saturated pixels). When necessary, movies were motion-stabilized using imageJ plugins. Endothelial structures were segmented in each image using a statistically-determined threshold value using Otsu’s method. Irrelevant artifacts such as single cells and small holes were then removed from the images by morphological “opening” of the image as well as its morphologically “closed” negative (using Matlab’s *bwareaopen* and *imclose* functions). All connected components were then isolated and catalogued using the *bwconncomp* function, and their “*perimetric complexity*” (defined as the squared contour length divided by the area, normalized by 4*pi) was calculated. Additionally, Each frame was processed via the Angiotool interface as described in the online user manual (see https://ccrod.cancer.gov/confluence/display/ROB2/Quick+Guide) to quantify vessel percentage area, total number of junctions, total vessels length, average vessel length, total number of end points and lacunarity.

## Additional Information

**How to cite this article**: Blinder, Y. J. *et al*. Vasculogenic dynamics in 3D engineered tissue constructs. *Sci. Rep*. **5**, 17840; doi: 10.1038/srep17840 (2015).

## Supplementary Material

Supplementary Information

Supplementary Movie S1

Supplementary Movie S2

Supplementary Movie S3

Supplementary Movie S4

Supplementary Movie S5

Supplementary Movie S6

Supplementary Movie S7

Supplementary Movie S8

Supplementary Movie S9

Supplementary Movie S10
