# Supplementary material for: Vasculogenic dynamics in 3D engineered tissue constructs
Source: Sci Rep. 2015 Dec 9;5:17840. doi: 10.1038/srep17840 (PMC4673462; doi:10.1038/srep17840)
Supplement: Supplementary Information [file srep17840-s1.pdf]

Vasculogenic dynamics in 3D engineered tissue constructs

Supplementary data

Yaron J. Blinder[1, 3], Alina Freiman[1], Noa Raindel[1], David J. Mooney[2,3], Shulamit Levenberg[1]

[1]Technion – Israel Institute of Technology, Israel

[2]School of Engineering and Applied Sciences, Harvard University

[3]Wyss Institute for Biologically Inspired Engineering at Harvard University

Supplementary figures:

S1 – Endothelial morphology with and without fibroblasts in culture after 7 days. Scale bar – 500µm**.**

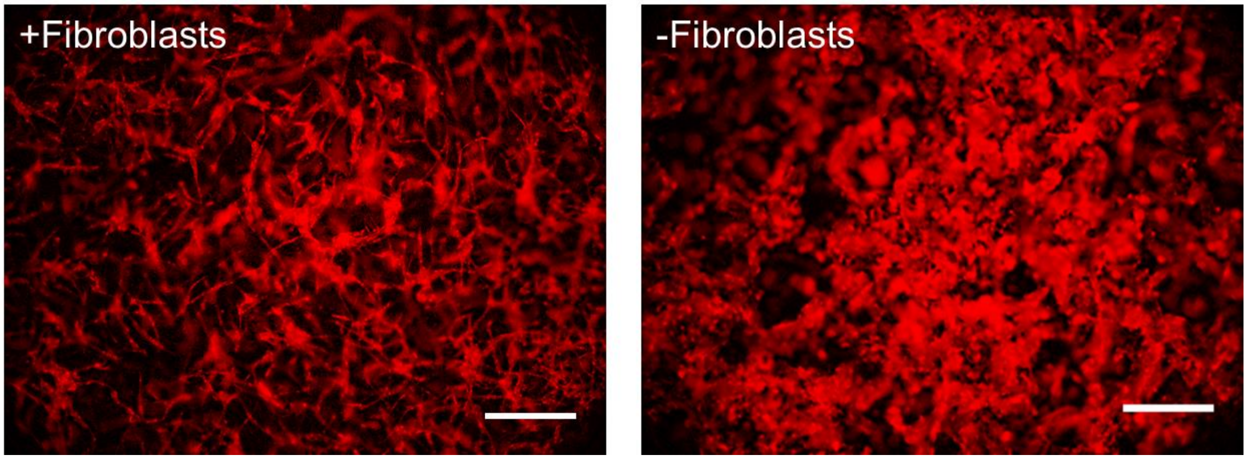

S2 - Quantification of network structure maturation by “Perimetric Complexity” metric.

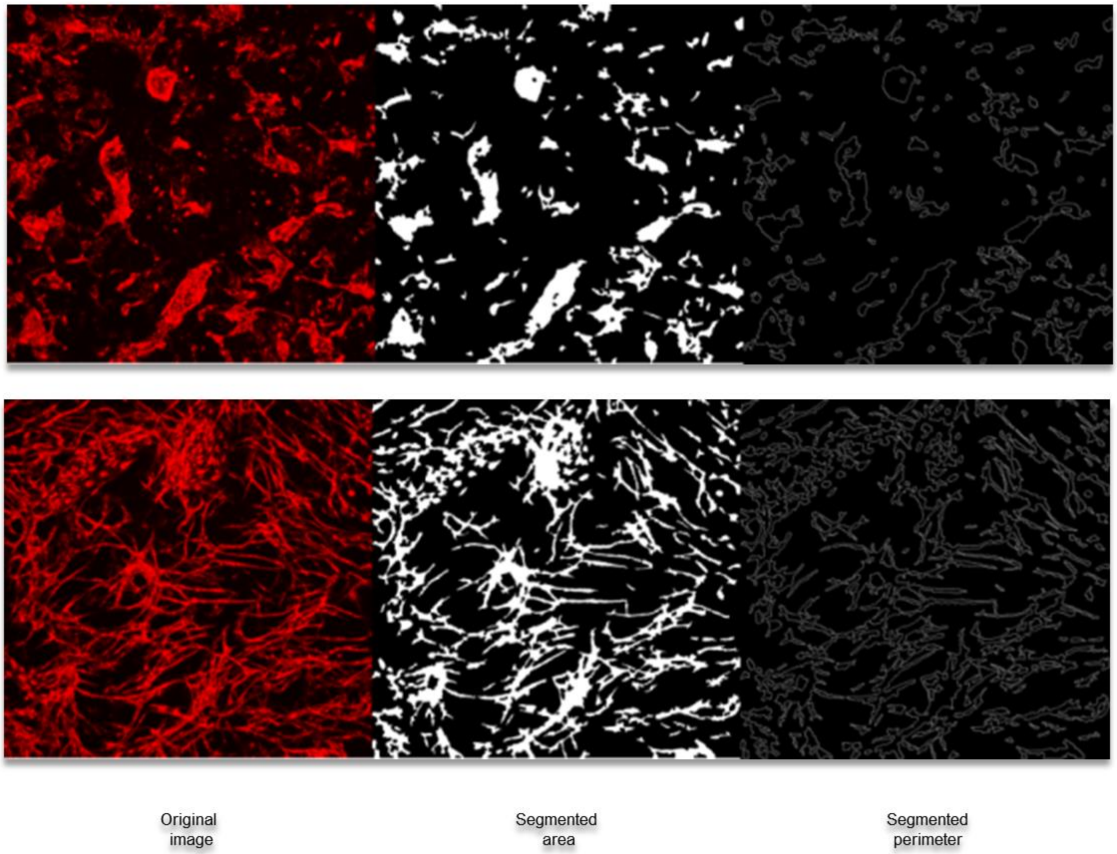

S3 - IHC of Collagen I expression over time. Scale bar – 250um.

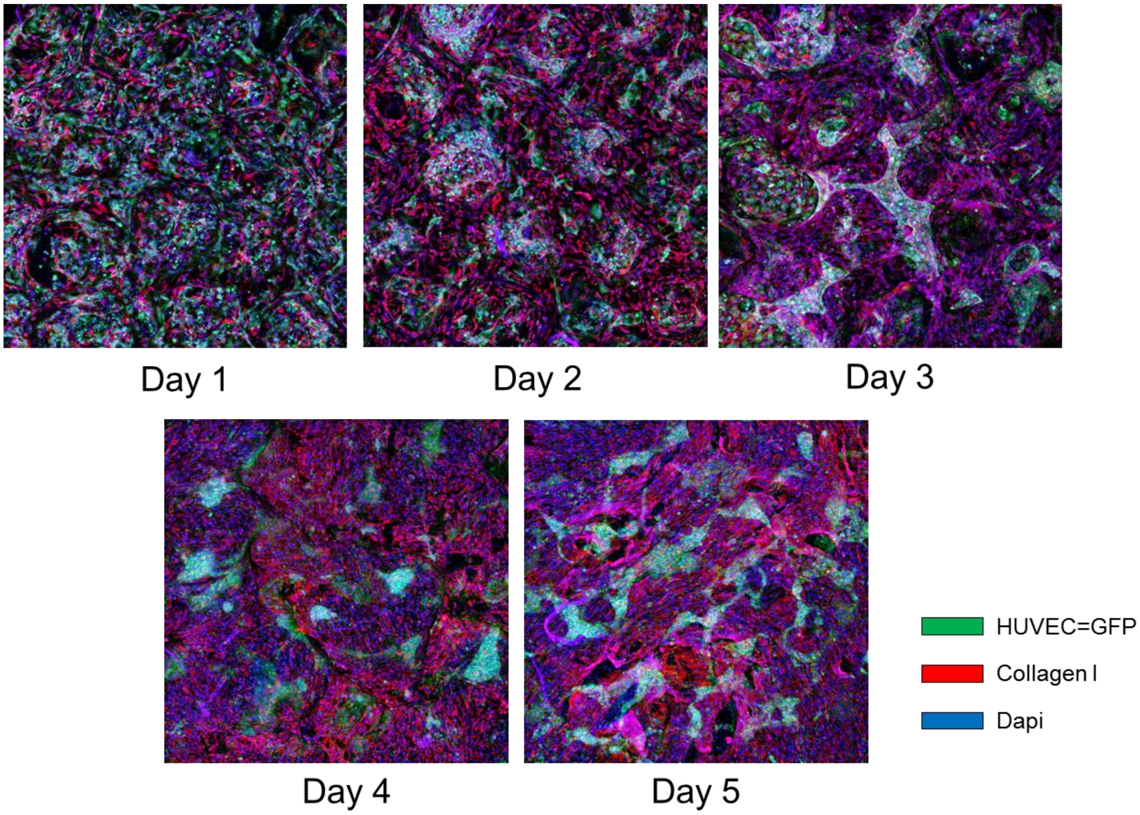

S4: IHC staining of scaffold cross-section for Collagen I and IV. A: GFP, B: Hoechst, C: Collagen IV., D: Collagen I, E: Overlay, F: 3D reconstruction (Imaris).

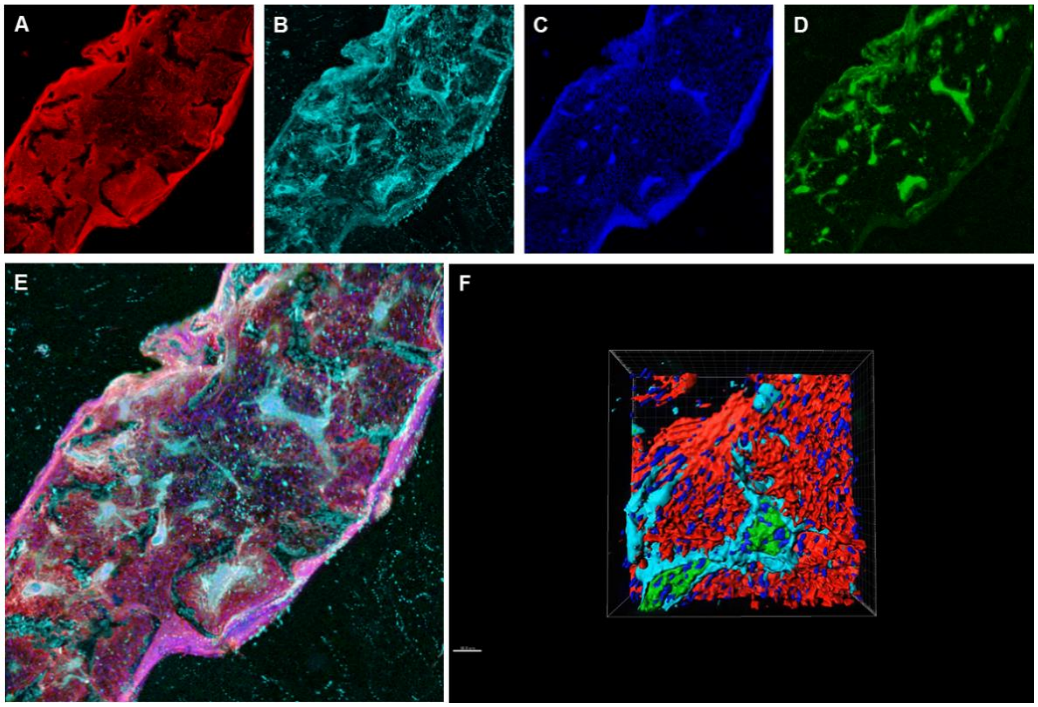

S5: IHC staining of scaffold cross-section for Laminin. A: Laminin, B: Hoechst, C: GFP, D: Merge. Scale bar = 100µm.

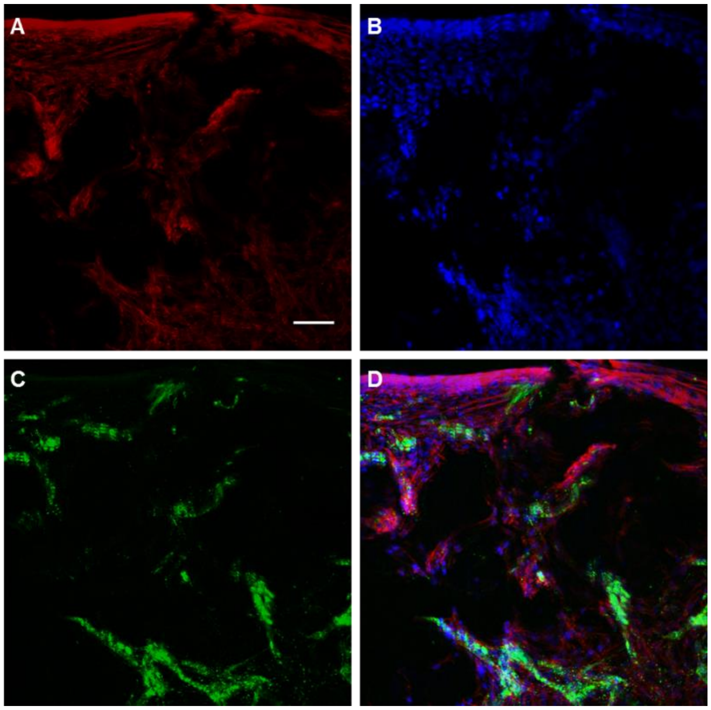

Supplementary movie captions:

M1 – Time lapse of endothelial and fibroblast morphogenesis in days 1-3 post seeding. HUVECs in red, HNDFs in green. Time step = 1hr.

M2 – Time lapse of endothelial morphogenesis in days 3-7 post seeding.

M3 – Time lapse showing an individual tip cell sprouting and displaying filopodia. Time step = 1hr.

M4 – Time lapse showing dynamic competition at the tip cell position. Time step = 1hr.

M5 – Time lapse showing junction formation through cluster thinning.

M6 - Time lapse showing junction formation through sprout anastomosis.

M7 –Time lapse movie of control vs. VEGFR2-inhibited neovascular morphogenesis.

M8 - Time lapse of neovascular formation side-by-side with its Angiotool segmentation.

M9 – Time lapse showing vessel regression and trimming in mature vascular constructs by VEGFR2 inhibition.

M10 - 3D reconstruction by IMARIS. Red – collagen I, Green – GFP HUVECs, Cyan – Collagen IV.
